# A cultural experience to support mental health in people aged 16–24 during the COVID-19 pandemic compared to a typical museum website: study protocol of an online randomised controlled trial

**DOI:** 10.1186/s13063-021-05441-z

**Published:** 2021-07-22

**Authors:** Rebecca J. Syed Sheriff, Matti Vuorre, Evgenia Riga, Andrew K. Przybylski, Helen Adams, Catherine J. Harmer, John R. Geddes

**Affiliations:** 1 Department of Psychiatry, University of Oxford, Oxford, UK; 2 Oxford Health NHS Foundation Trust, Oxford, UK; 3 Oxford Internet Institute, University of Oxford, Oxford, UK; 4 Gardens, Libraries and Museums Division, Ashmolean Museum, University of Oxford, Oxford, UK; 5 NIHR Oxford Health Biomedical Research Centre, Oxford Health NHS Foundation Trust, Oxford, UK

**Keywords:** Depression, Anxiety, Youth, Experimental medicine, Randomised controlled trial, Culture, Web-based intervention

## Abstract

**Background:**

Despite the high prevalence of common mental disorders in adolescents and young adults, and their association with poor health and socio-economic outcomes throughout the lifespan, many young people do not seek or receive help for such disorders. There is growing interest in the community sector in supporting mental health in young people; however, there is little by way of experimental research in this area. During the COVID-19 pandemic and lockdown, we designed an online cultural experience to reduce anxiety and depression and support mental health in people aged 16–24.

**Methods/design:**

The O-ACE POP (Online Active Community Engagement Proof of Principle) study is a UK-based online randomised controlled trial of an online cultural experience named Ways of Being, involving human centred narratives and viewpoints, compared with a typical museum website (the Ashmolean Museum). We aim to compare efficacy on  affect,  symptoms of epression and anxiety, flourishing and loneliness as well as investigating potential mechanisms of action.

**Discussion:**

The COVID-19 pandemic has provided a unique opportunity to design an innovative approach to supporting mental health in young adults. Findings derived from this study will allow us to evaluate the efficacy of this intervention and will inform the design of studies to further refine the resource and test it further.

**Trial registration:**

ClinicalTrials.gov NCT04663594. Registered on 11 December 2020 (submitted in same form 27 November 2020). Protocol v1.0: 27 November 2020. Date recruitment began: 4 December 2020. Recruitment complete (estimate): February 2021

## Background

Despite the high prevalence of the common mental disorders, anxiety and depression, early in life [1] and their association with poor mental and physical health and socio-economic outcomes throughout the lifespan [2–4], many young people do not seek or receive help for such disorders [5–7]. Moreover, there is evidence that rates of mental disorders are rising in adolescents and young adults [8], and even those who have sought help are at high risk of discontinuing their contact with mental health services [9]. Thus, there is increasing interest in the community sector in supporting mental health in young people; however, there is little by way of experimental research in this area [5].

In the context of the COVID-19 pandemic, economic uncertainty and feelings of isolation brought about by social distancing measures have meant that mental health and wellbeing have become a major public health concern. An epidemiological study compared the proportion of the population of Great Britain with depression during the COVID-19 pandemic (June 2020) to previous to the pandemic (July 2019–March 2020). Young adults (aged 16 to 39 years) were more likely than other adults to experience depressive symptoms during the COVID-19 pandemic. Around one in three young adults experienced depressive symptoms (moderate-severe) during the COVID-19 pandemic compared with about one in nine previously [10].

Intervention research has focused on treatments administered by mental health professionals [5], the mainstay of which are medications and talking therapies. However, these may not be accessible, acceptable or appropriate for the majority of young people with mental health problems [2]. In this context, there is mounting interest in the community sector in delivering interventions for youth mental health, such as museums, libraries and outdoor activities. In this context, we conducted thorough searches for experimental studies of community resources for anxiety or depression (https://www.crd.york.ac.uk/PROSPERO/display_record.php?RecordID=204471). We were unable to identify any trials investigating museums or online museums to treat, reduce or prevent anxiety and depression in young adults. This is consistent with a systematic review which sort to identify studies of strategies not accompanied by a mental health professional for anxiety and depression in children and young people, in which no studies of museums or online museums, were identified [5].

We have designed O-ACE POP (Online Active Community Engagement Proof of Principle Study), a randomised controlled trial, to investigate the efficacy and potential mechanisms of action of an innovative online museum experience to reduce anxiety and depression and support mental health in people aged 16–24, compared with a typical museum website (Table 1).

**Table 1:** WHO checklist

Primary registry and trial identifying number	https://clinicaltrials.gov NCT04663594
Date of registration in primary registry	11 December 2020
Secondary identifying numbers	N/A
Source(s) of monetary or material support	University of Oxford COVID-19 Research Response Fund, the Westminster Foundation and the Huo Family Foundation
Primary sponsor	University of Oxford COVID-19 Research Response Fund
Secondary sponsor(s)	The Westminster Foundation
Contact for public queries	Rebecca.sheriff@psych.ox.ac.uk
Contact for scientific queries	Rebecca.sheriff@psych.ox.ac.uk
Public title	Online Culture for Mental Health in People Aged 16–24 (O-ACE POP)
Scientific title	A Cultural Experience to support Mental Health in people aged 16–24 during the COVID-19 pandemic compared to a typical museum website
Countries of recruitment	Online trial (based in the UK but no exclusion of participants from overseas)
Health condition(s) or problem(s) studied	Depression and anxiety
Intervention(s)	The Ashmolean Website Ways of Being (Web Experience)
Key inclusion and exclusion criteria	People aged 16–24 (or 18–24 if outside the UK) Exclusion: No access to a laptop or desktop computer with recent browser
Study type	Randomised controlled trial
Date of first enrolment	4 December 2020
Target sample size	400
Recruitment status	Ongoing
Primary outcome(s)	Mood (PANAS) K10 (Kessler Psychological Distress Scale)
Key secondary outcomes	Flourishing, loneliness, Facial Expression Recognition Task, Probabilistic Incentive Learning Task

## Preparatory work

During the first lockdown in the UK, many people turned to online content provided by museums and other cultural organisations . We conducted an online survey into the potential mental health benefits of online cultural resources. The self-reported mental health benefits of online culture were more marked in regular users of online culture and differed with age group. People aged 16–24 were less likely to be regular users of online culture, but echoing population studies [10], higher proportions had clinically significant levels of distress, signifying probable anxiety or depression. We therefore surmised that targeting by age group might maximise the benefits of online culture.

We then utilised qualitative research methodologies to investigate the potential mental health benefits of cultural resources in  young adults. We used this research along with co-production with young adults to design and produce an online cultural experience to reduce distress and support mental health in people aged 16–24. This led to the production of Ways of Being (WoB), a cultural web-experience used as an intervention in this trial. These studies will be presented and reported separately.

## Methods/design

**Fig. 1:**
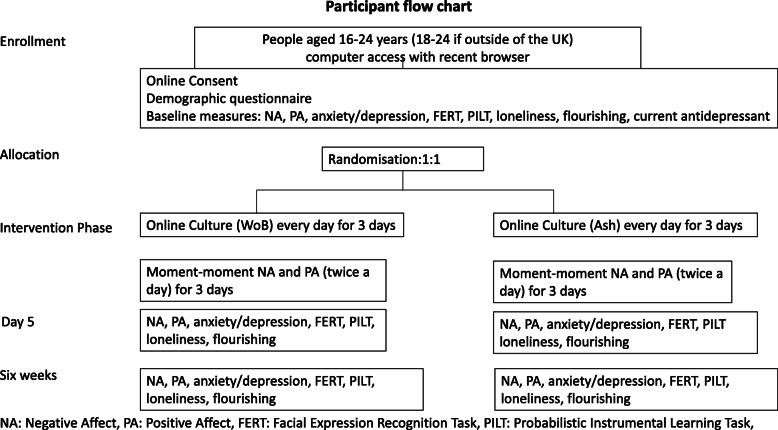
Participant flow

The O-ACE POP study is a randomised controlled trial, comparing an online cultural web experience designed to reduce distress and promote positive mental health in people aged 16–24 and a typical museum website. The participant flow chart is shown in Fig. 1.

## Study objectives

1. To compare the effectiveness of the Ways of Being (WoB) web experience with the Ashmolean Museum Website on the primary outcomes of negative and positive affect and psychological distress and secondary outcomes, flourishing and loneliness
2. To explore the potential mechanisms of action of online culture using online cognitive tasks, the Facial Expression Recognition Task (FERT) and the Probabilistic Instrumental Learning Task (PILT)

## Study design

The trial is designed as an online prospective, parallel group, exploratory randomised controlled trial. Eligible participants will be randomised in a 1:1 allocation to WoB or the Ashmolean Website, directing them to the Ashmolean from home collections (please see Fig. 1 for participant flow).

## Ethics

Online consent procedures will be followed. Ethics approval was provided by the University of Oxford Central University Research Ethics Committee (CUREC), approval reference number R70187/RE007. Any protocol amendments will be communicated to the REC, and any changes will be applied after the relevant ethics approval has been obtained. On the consent form, participants will be asked if they give permission to use anonymised data in future studies and to share data with other researchers (e.g. in online databases). They will also be asked if they give permission to be contacted for interviews or focus groups.

We followed ethical guidance and advice regarding consent procedures for this age range and are able to include people aged 16 and 17 years as competent youths. However, this category cannot be applied to those currently outside of the UK due to potential legislative differences.

## Participants and recruitment

People aged 16–24 years (16–24 if based in the UK or 18–24 if based overseas) with access to a desktop or laptop computer running a recent browser (Edge or Chrome on Windows, and Safari or Chrome on a Mac) will be eligible for this study.

We will actively recruit people of the target age through social media (Facebook and Instagram), student organisations (e.g. Student Unions) and schools (e.g. via the online publication, The Day, an online news service for use in schools, colleges and homes) with a link to participant information and E-consent procedures for those who fulfil inclusion criteria and consent to entering their email address.

Consenting participants will enter a demographic questionnaire including information regarding country, age, gender, ethnicity, isolation status, income, relationship status, use of online culture, education, current employment, current and previous mental health and COVID-19 status. Participants will then be emailed a unique ID number and requested to complete a baseline assessment on a computer, including self-report measures, current antidepressant use and online tasks. They will then be randomised to WoB or the Ashmolean. Participants will be able to continue with other treatments for anxiety and depression as well as any other activities or interventions that they use to support their mental health. They will be asked about current antidepressant use and help-seeking.

**Fig. 2:**
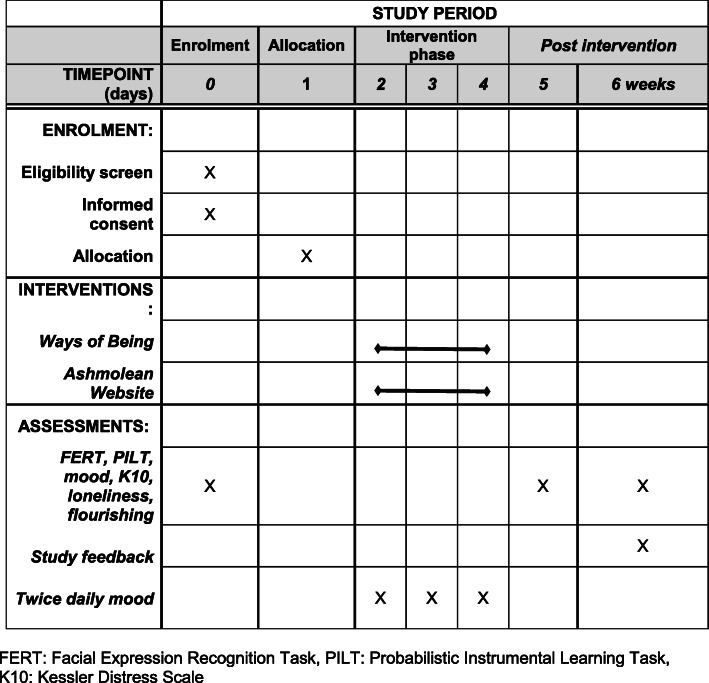
Schedule of enrolment, interventions, and assessments. FERT, Facial Expression Recognition Task; PILT, Probabilistic Instrumental Learning Task; K10, Kessler Distress Scale

Participants will have the option of consenting to text messaging to remind them to complete the intervention and measures. There will also be email reminders to complete the measures at day 5 and 6 weeks. In addition, participants will be offered Amazon vouchers as a thank you for their time at certain timepoints. Clear information regarding the time taken to complete questionnaires and tasks at each timepoint are clearly stated in the information for participants (see Table 2) (Fig. 2).

**Table 2:** Overview containing schedule and estimated times for completion of each stage provided in information for participants and at key points throughout the trial

Day	What happens	e-Voucher
Baseline/day 1	Informed e-consent procedure (link at bottom of this information page) Demographics questionnaire following consent page (~ 10 min) Email with a unique Participant ID (PID) and link to online platform for all further tasks and assessments. Baseline assessment (~ 30–40 min, must be on a computer) Email to inform you of allocation (*WoB or #Ash)	
Day 2	Morning mood (*WoB or #Ash) Evening Mood (~ 5 min, on device of choice)	
Day 3	Morning mood (*WoB or #Ash) Evening Mood (~ 5 min, on device of choice)	
Day 4	Morning mood (*WoB or #Ash) Evening Mood (~ 5 min, on device of choice)	
Day 5	Exit Assessment (~ 30-40 min, must be on a computer)	£30
6 weeks	Follow-up Assessment (~ 30-40 min, must be on a computer)	£10

## Interventions

Ways of Being (WoB) is an online cultural experience designed to reduce symptoms of anxiety and depression and support mental health in people aged 16–24. Based on the findings of qualitative research with people of the target age range, human centred narratives and viewpoints are a focus of the experience. WoB was designed and produced via co-production with young people and will be described in full in a separate publication. The experience centres around the objects and artworks of the Ashmolean Museum in Oxford and partner museums. The experience combines text, audio and aesthetic elements. Each participant will be given a unique ID to log in to WoB.

The Ashmolean Website is a generic museum website owned by the Ashmolean Museum of Art and Archaeology at the University of Oxford. The Ashmolean is the oldest public museum in the UK and has rich and diverse collections from around the globe, ranging from classical civilisation to the Pre-Raphaelites and modern art. Participants will be directed to the Ashmolean from home collections.

Participants from both arms will be asked how much time they have spent on their allocated intervention. This will include self-report items on their use during the 3-day intervention phase and subsequent use in the 6-week follow-up period.

## Measures

### Primary outcomes

#### The Positive and Negative Affect Schedule

The Positive and Negative Affect Schedule (PANAS) is a widely used scale of emotion and comprises 20 items, 10 measuring positive affect (excited, inspired, etc.) and 10 measuring negative affect (upset, afraid, etc.), on a Likert Scale of 1 (very slightly) to 5 (extremely) [11]. This will be asked with reference to the previous seven days at baseline and 6-week follow-up, the previous 3 days at day 5 and at that precise moment twice daily during the 3-day intervention phase. The PANAS is a reliable and valid measure of depression and anxiety in young adults. The PANAS was chosen for this study as it is relatively stable but also sensitive to mood fluctuations if used with short-term instructions (e.g. now) [11]. The utility of this measure is enhanced by the provision of large-scale normative data in the general UK population [12]. The basic psychometric data for the PANAS was developed from studies in undergraduates, of a similar age range as the participants of this current study, and it is well validated in young adult populations [11].

In previous studies, negative and positive affect have emerged consistently as two dominant and relatively independent dimensions in the structure of affect. The Negative Affect (NA) scale of the PANAS is highly internally consistent, largely uncorrelated, and stable (Cronbach alpha reliabilities for intercorrelations and internal consistency range from .84 to .87) [11]. NA reflects the degree of subjective distress arising from various negative mood states, such as guilt, anger, fear and nervousness. High NA is related to self-reported stress and difficulty coping with negative events. The 10-item Positive Affect (PA) scale is valid and reliable with high internal consistency. Reliabilities range from .86 to .90 [11]. High PA is characterized by energy, concentration and engagement [11].

#### Psychological distress

The K10 is a well validated self-report measure of psychological distress [13]. The K10 comprises ten questions inquiring about the frequency of depressive and anxiety symptoms over the previous 4 weeks. The K10 has been used as an epidemiological measure to screen for clinically significant depression and anxiety in community samples worldwide. The K10 strongly discriminates between community cases and non-cases of mental disorders identified by a structured clinical interview [14]. In addition, it has been used as an outcome measure in intervention studies [13]. The K10 will be asked with reference to the previous 4 weeks at baseline and 6-week follow-up, and with reference to the previous 3 days at day 5. The K10 is a reliable measure with all items of relevance to young people of this age range [15].

### Secondary outcomes

#### Flourishing

The Flourishing Scale is an 8-item measure of self-perceived success in domains such as self-esteem, relationships, optimism and purpose [16]. It is validated in community samples of young people internationally [17] and provides a single psychological well-being score.

#### Loneliness

The UCLA Loneliness Scale is a self-report inventory that uses a Likert-type scale to assess subjective feelings of loneliness [18]. There is a validated three-item version [19] to which we have added a direct measure of loneliness as recommended by the Office of National Statistics for measuring loneliness in those aged 16 years and over [20].

### Online cognitive tasks

#### Facial Expression Recognition Task

The Facial Expression Recognition Task provides an objective measure of emotional bias which is associated with vulnerability to depression [21]. Facial expressions of anger, fear, disgust, sadness, surprise, are presented across different intensity levels (0–100%, in 10% steps) and participants are asked to indicate the facial expression that they can see. Two hundred fifty trials are presented split into separate blocks. Accuracy, reaction time and misclassifications are computed in this task. Low mood is associated with an increased tendency to pick up on negative facial expressions compared to positive ones [21]. This testing and analyses will be supported by the infrastructure of the NIHR Oxford Health Biomedical Research Centre

#### Probabilistic Instrumental Learning Task

The Probabilistic Instrumental Learning Task (PILT), adapted for use online, is based on the Instrumental Learning task described by Pessiglione et al. [22]. We chose this task as depression is characterized by deficits in reward-based decision making. In particular, reward sensitivity is thought to relate to anhedonia, a symptom seen in depression which reflects a deficit in the ability to experience pleasure. We hypothesised that the mechanism of action of cultural experiences on depression might involve an improvement in reward processing and therefore changes in the performance of this task may signify changes in the vulnerability to depression relating to a cultural intervention. This could provide vital clues as to the underlying mechanism by which such interventions might work on a neural level.

The PILT has been described in full elsewhere [23, 24]. In brief, the participant gains or loses points by choosing between two paired images. Participants are required on each trial to choose one of two pairs of symbols. One pair is associated with win outcomes (win 20 pence or no change) and the other with loss outcomes (lose 20 pence or no change). Each symbol in the pair corresponds to reciprocal probabilities (0.7 or 0.3) of the associated outcomes occurring. Participants are instructed to pick the symbol they believe is most likely to win (or least likely to lose), with the aim of maximising their monetary pay off. Feedback on the outcome of each trial is given after a choice is made. Participants complete two runs of the task, each with a new set of four symbols. The total amount won, total amount lost, end total, symbol choice and choice consistency are recorded. The explicit aim is to maximise points.

### Trial feedback

At the 6-week follow-up, we will include a research experience survey, based on the NIHR Research Experience checklist (https://www.nihr.ac.uk/documents/optimising-the-participant-in-research-experience-checklist/21378?diaryentryid=60465). This survey includes 8 items asking participants to rate various aspects of their research experience on a Likert scale (1—strongly disagree to 5—strongly agree).

### Power analysis and sample size

We aim to recruit over 400 participants. We approximated the statistical power to detect a significant effect of WoB on PANAS scores by assuming an effect size of 0.3 standard deviations of group (WoB vs. Ashmolean Website). With 200 participants in each group, and no pre-intervention differences, a Welch’s t test would have 85% probability of rejecting the null hypothesis under these parameters.

### Randomisation and blinding

Participants will be randomized using computer generated random sequencing, in blocks of six stratified by gender with an equal allocation ratio*.* The allocation sequence is to be configured by a data manager blinded to the allocation group. The participants will be allocated by a researcher blinded to all study data except gender during the entire randomisation and allocation procedure. The participants will be aware of which intervention they have been allocated to. The interventions as well as all measures are online and self-report.

### Data management

The Gorilla Experiment Builder (www.gorilla.sc) is a cloud-based research platform that allows researchers to design and administer behavioural (reaction-time) experiments online.

The Gorilla database is encrypted. The investigators own the data that has been collected using Gorilla as well as the data. The investigators can generate and access the anonymized data from Gorilla. Data containing personal identifiable information (e.g. email addresses) are stored in a separate database from the anonymised research database; both hosted by Gorilla. Participants who do not complete measures within a reasonable timeframe from allocation will be timed out of the research database. Completed data will be downloaded and safely stored for statistical analysis on the University of Oxford Study drive.

### Analysis plan

A descriptive analysis will be performed for the whole population and each group (WoB and Ashmolean Website). Categorical variables will be described, presenting the numbers and frequency of each. Quantitative variables will be described using usual positional and dispersion parameters.

We will perform an intention to treat analysis to include all participants randomised with any outcome data. Participants will continue to be invited for assessments whether or not they use the allocated intervention. Missing item responses are not possible by design. Missing data, if any, will happen due to participants discontinuing with outcome assessments. However, their data for timepoints prior to that will still be included in the multilevel model, so no additional methodological steps are required regarding missing data.

For NA and PA we will separately regress the subscale means on baseline score (Day 1 measurement), an indicator for Group (Ashmolean website / WoB), and contrast code age (16-17 / 18-24), sex (Male / Female), ethnicity (White / Other), relationship status (In / Not in relationship or other), and current antidepressant use (Yes / No). We will include random intercepts for participants. As there are multiple timepoints at which the group comparison could be made, we will allow the Group effect to vary as a random effect across the measurement timepoints. We will perform a similar analysis for the K10, however as there are fewer measurement timepoints for the K10 compared to the PANAS (2 vs 8) we treat time points as fixed effects. This is the same for secondary outcomes, flourishing and loneliness. The 6-week measures are primarily included to assess whether early differences in the performance of cognitive tasks predict changes in the primary outcomes at 6 weeks.

We will conduct subgroup analyses based on age group (16-17 or 18-24), sex, ethnicity, baseline mental disorder, antidepressant use and previous regular use of online culture (more or less than once a month). We hypothesise that mental disorder at baseline, those not on antidepressants and regular users of culture would be more likely to demonstrate group differences.

### Embedded qualitative study

An embedded qualitative study will be conducted in order to ascertain participants experience of the interventions and research process to aid interpretation of the quantitative data and ascertain their views regarding the optimal use of the interventions and further testing. More specifically participants will be asked about the following:

Experience of the intervention

- Helpfulness to mental health
- Adverse or unintended consequences
- Optimal time of use
- Suggestions for improvement for mental health

Experience of the research

- How the process of participation in the study was experienced
- How the testing was experienced
- Suggestions for improvement for the research process and/or testing

Participants who have consented for further contact will be selected purposively and iteratively. Baseline data will be used to ensure heterogeneity by sociodemographic characteristics, mental health status and previous use of online culture. Participants will be emailed to invite them to consent for a focus group. Focus groups will be conducted separately for each intervention arm at a time convenient to the participants via Zoom. These will be recorded and transcribed for analysis by a multidisciplinary team with expertise in qualitative methodologies. We aim for at least 12 participants to be included in focus groups. When assessing thematic saturation, the sample size will be reviewed [25].

At the 6-week follow-up, there will be free text items regarding what was positive about the research experience and what could be improved. There will also be items relating to the experience of using the allocated intervention and how its use could be optimised for mental health, including two free text options, ‘Please describe any noticeable positive or negative impacts of your allocated cultural experience’ and ‘Are there any ways in which the allocated intervention could be improved for mental health, in your opinion?’

Transcripts and free text responses will be analysed separately. Both transcripts and free text responses will be coded using specialist computer software, NVIVO [26]. Data analysis will take a sequential-explanatory approach [27]. We will combine deductive and inductive methods to investigate whether the theory developed in the preparatory phase holds true, but we will also be open to emerging themes. Analytic meetings will be held with the research team to discuss and optimise validity. Attention will be paid to integrating quantitative and qualitative aspects for evaluating the trial and its interventions [28].

### Monitoring

The study will be overseen by a Trial Steering Committee. The Trial Steering Committee will meet regularly and at key stages of the trial, at a minimum at planning, pre-recruitment and prior to completion. The Project Management group will meet at least weekly for the duration of the trial. Any data-related issues, should they arise will be discussed within the Project Management group and with the Trial Steering Committee at the next meeting, or meetings will be expedited if deemed necessary. Access to study data will be provided to authorised representatives from the Sponsor for monitoring and audit purposes if required.

The Trial Steering Committee consists of the Head of Department and researchers from the University of Oxford Department of Psychiatry, a Consultant Psychiatrist, two staff members from Oxford University Gardens, Libraries and Museums, three Public and Patient involvement (PPI) members, researchers from the Oxford Internet Institute (including the Director of Research) and three students and members of staff from the target age range. The Trial Steering Committee decided that a separate Data Monitoring Committee would not be necessary based on the short time frame of the intervention phase and low risk nature of the trial.

Both interventions and trial procedures relevant to the first 5 days of the study have already been trialled on eleven volunteers and detailed feedback taken and discussed in detail by the Trial Steering Committee. This is a low-risk study, and we do not anticipate any reasons for unblinding; therefore, study-specific interim analyses, stopping or unblinding procedures have not been put into place. Should the need arise, the relevant University of Oxford standard operating procedures will be followed.

## Discussion

O-ACE POP has two main goals. The first is to assess the effectiveness of a web-based cultural experience tailored to support mental health in young people compared with the Ashmolean Museum Website, on measures of mood, as well as loneliness and flourishing. The second is to investigate the impact on objective measures (cognitive tasks) predicting vulnerability to common mental disorders. Young people identified that human based narratives and other people’s viewpoints would have the potential to reduce distress, improve their mental health and enhance feelings of connection with others. This study will allow us to examine whether an intervention based directly on the opinions and experiences of young people and targeted at them has the potential to work over and above what is already available (without these specific features). This study will allow us to explore who, how and if online cultural experiences are effective for the mental health of young people.

Completing this study in this age range has many important advantages. First, given the trajectory of common mental disorders, this is a critical timepoint for minimising their impact in terms of disease burden, lifetime distress, illness and social and economic factors such as lost productivity. Second, this is a peak period for development of common mental disorders and, thus, a critical timepoint for prevention. Third, research in this age range is less likely to be confounded, for example by multiple episodes and medical or psychological interventions.

## Strengths

Many now advocate the engagement of young people, co-production and an increased evidence base for community interventions for CMDs in young people [5]. This trial is the first of its kind, and whilst ambitious in its endeavour, there are no previous trials of online museums for mental health in young people on which to build. We worked closely with young people of the target age-range to co-produce the web experience and with PPI of the target age range to design the experiment. We have kept broad inclusion criteria so as not to exclude people who could potentially benefit and use subgroup analyses to generate hypotheses as to the optimal use of these interventions, for investigation in further studies. We also piloted the study and intervention on eleven volunteers in order to assess smooth running of the procedures and identify any potential unintended or adverse effects. On detailed discussion amongst the study team and trial steering committee, given the feedback from volunteers, the nature of the interventions and length of the intervention phase, the risk of adverse effects were deemed minimal.

## Limitations

Due to technical aspects of the cognitive tasks and the WoB web-experience, for inclusion into this study participants require access to a computer (laptop or desktop) with a recent Web Browser. This may mean that some groups may not access this study or may not fulfil the inclusion criteria. It is difficult to assess how many people this may apply to. However, internet access in general has increased in recent years. In 2020, 96% of households in Great Britain had internet access, an increase from 93% in 2019 and 57% in 2006 [29]. Many of the barriers to internet use involve not wanting to use the internet or concerns regarding security [30]. It is not clear from the literature how many people in this specific age range would not have access to a computer with a recent browser and how this relates to their mental health need.

## Implications

Studies that attempt to quantify the potential benefits of community resources for mental health may aid policy makers to make informed decisions regarding allocation of limited resources. In addition, the accessibility of technological resources for using, optimising or testing the mental health benefits of community resources is a major consideration for which evidence is scarce. For example, further generations of optimised community webtool interventions and perhaps even the platforms for testing of their mental health benefits may become possible on Smartphones or Tablets, thus increasing the accessibility of such interventions and studies to people from lower income groups who may have reduced access and potentially greater mental health need. In addition, this study develops rigorous testing to methodologies which encompass engagement with young people and co-production of engaging and acceptable interventions.

The COVID-19 pandemic and associated restrictions has been detrimental to the mental health of the population but has provided a unique opportunity to design an innovative approach for mental health in young adults*.* Findings derived from this study will allow us to evaluate the efficacy of these interventions and will lay the groundwork for the design of studies to further enhance, refine and test online and in-person cultural resources for mental health in target populations. Moreover, it will allow us to identify whether online experimental studies in the area of community resources for mental health in young people are a fruitful avenue for research.

## Data Availability

The authors will have access to the study data. We plan to make the dataset and coding used for the analysis available on the Open Science Framework.
